# Enhanced Adsorptive Bioremediation of Heavy Metals (Cd^2+^, Cr^6+^, Pb^2+^) by Methane-Oxidizing Epipelon

**DOI:** 10.3390/microorganisms8040505

**Published:** 2020-04-01

**Authors:** Muhammad Faheem, Sadaf Shabbir, Jun Zhao, Philip G. Kerr, Nasrin Sultana, Zhongjun Jia

**Affiliations:** 1 State Key Laboratory of Soil and Sustainable Agriculture, Institute of Soil Science, Chinese Academy of Sciences, Nanjing 210008, China; faheem_127@yahoo.com (M.F.); zhaojun@issas.ac.cn (J.Z.); nasrinjc_sau@yahoo.com (N.S.); 2 University of Chinese Academy of Sciences, Beijing 100049, China; 3 College of Environment, Hohai University, Nanjing 210098, China; sadaf.dar83@yahoo.com; 4 School of Biomedical Science, Charles Sturt University, Wagga Wagga, NSW 2678, Australia; philip.kerr@gmail.com

**Keywords:** epipelon, heavy metals, bioremediation, methane oxidation, DNA-SIP

## Abstract

Cadmium (Cd), chromium (Cr) and lead (Pb) are heavy metals that have been classified as priority pollutants in aqueous environment while methane-oxidizing bacteria as a biofilter arguably consume up to 90% of the produced methane in the same aqueous environment before it escapes into the atmosphere. However, the underlying kinetics and active methane oxidizers are poorly understood for the hotspot of epipelon that provides a unique micro-ecosystem containing diversified guild of microorganisms including methane oxidizers for potential bioremediation of heavy metals. In the present study, the Pb^2+^, Cd^2+^and Cr^6+^ bioremediation potential of epipelon biofilm was assessed under both high (120,000 ppm) and near-atmospheric (6 ppm) methane concentrations. Epipelon biofilm demonstrated a high methane oxidation activity following microcosm incubation amended with a high concentration of methane, accompanied by the complete removal of 50 mg L^−1^ Pb^2+^ and 50 mg L^−1^ Cd^2+^ (14 days) and partial (20%) removal of 50 mg L^−1^ Cr^6+^ after 20 days. High methane dose stimulated a faster (144 h earlier) heavy metal removal rate compared to near-atmospheric methane concentrations. DNA-based stable isotope probing (DNA-SIP) following ^13^CH_4_ microcosm incubation revealed the growth and activity of different phylotypes of methanotrophs during the methane oxidation and heavy metal removal process. High throughput sequencing of ^13^C-labelled particulate methane monooxygenase gene *pmoA* and 16S rRNA genes revealed that the prevalent active methane oxidizers were type I affiliated methanotrophs, i.e., *Methylobacter*. Type II methanotrophs including *Methylosinus* and *Methylocystis* were also labeled only under high methane concentrations. These results suggest that epipelon biofilm can serve as an important micro-environment to alleviate both methane emission and the heavy metal contamination in aqueous ecosystems with constant high methane fluxes.

## 1. Introduction

Heavy metals and the global rise in methane level both are serious threats to the environment and humans directly or indirectly [1,2]. According to the National Oceanic and Atmospheric Administration (NOAA) of USA, global methane emission has increased up to 1858 ppb till 2019, compared to pre-industrial times (722 ppb), and its impact on the environment has been 34 times greater than CO_2_ over a period of 100 years, as stated by the Environmental Protection Agency (EPA). It thus poses great challenges to address global warming and climate change by optimizing and developing the strategies for the reduction of the average level of global methane emissions, considering that it traps 21 times more heat than CO_2_ [3].

Wetland is an important target of methane reduction contributing approximately 20% of the global methane budget, while organic and inorganic sources of water pollution resulted in 1.8 million deaths during the year 2015 [4]. Heavy metals have emerged as a serious water contaminant in the present scenario as they tend to accumulate in the human body and result in mutagenic, teratogenic and carcinogenic changes [5,6]. According to the US-EPA, lead (Pb), cadmium (Cd) and chromium (Cr) are designated as the top priority contaminants that are of major public concern [7]. Numerous studies have been focused on physico-chemical approaches, such as ion exchange, flocculation, membrane filtration, biosorbents, electrodialysis, reverse osmosis and precipitation, for heavy metal removal in recent decades [8,9,10]. Though some of these techniques are effective in the removal of heavy metals, practical applications are not easily employed on a large scale due to their high cost and the secondary contaminations by the chemicals used in the processes. Several biological methods such as immobilization by biochar and rice straw, hyper accumulator plants, specific functional microbes have already been reported for heavy metals removal [11,12]. However, they require high labor cost and are not fully suitable in many cases under in situ conditions. Therefore, there is a need for a cost-efficient and environmentally sustainable technology that can remove heavy metals and methane gas simultaneously.

Methanotrophs have emerged as a tool of bioremediation due to the presence of methane monooxygenase (MMOs: pMMOs and sMMOs) enzymes with a distinctive capability of utilizing a vast array of compounds [13], including aliphatic halides and aromatic derivatives. The unique chemical reactivity and comprehensive substrate profiles of methane monooxygenases represent its potential implementation in bio-remediation of heavy metals [14].

In the last decade, a few studies have described the removal of heavy metals by methanotrophs. *Methylosinus*, a type II methanotrophs contributed 11–21% of the total bacterial DNA in the biofilm after addition of chromium (Cr) metal [15]. Additionally, the presence of Hg(II) and As(V) reductases was also confirmed in almost eight genera of methanotrophs, demonstrating that the metabolic potential of methanotrophs is generally overlooked [16]. *Methylococcus capsulatus* (Bath) was found to effectually bioremediate aquatic pollution caused by a broad range of chromium(VI) concentration (1.4–1000 mg L^−1^ of Cr^+6^) [17]. However, these methods took a longer time duration for the removal of heavy metals (20–90 days), moreover, the taxonomic identities of active methanotrophs remain poorly understood during the removal of heavy metals in complex aquatic environment.

Epipelon are periphytic biofilms that are known to promote the bio-stabilization of sediments and regularize benthic-pelagic nutrient cycling in benthic-pelagic zones of marine environments [18]. Periphyton are also known as an in situ strategy that has a promising role to stabilize micro-ecosystem, as well as in the restoration of devastated surface water ecologies [19] while the complex microbial communities and porous structures within can retain heavy metals [20]. Many microbial communities in periphytons are capable of adsorbing heavy metals by the secretion of extra polymeric substances (EPS) from cyanobacteria, green algae, diatoms and a variety of other bacteria [21]. Due to the intricate community structures, periphytic biofilms have a high resilience and capability to habituate to a wide range of concentrations of heavy metals and can maintain sustainable metabolic activities [22,23]. Periphytic raw material is also readily available and has a high metal removal and sorption potential, due to functional polar groups that are abundantly found on the periphytic surface [24], whereas, sludge after pollutant sorption, is less effective when compared to all other treatments. The presence of methanotrophs in lake sediments, as well as in wetland periphytons, has already been reported [25], but the methane oxidation capability of these biofilms have yet to be explored and the information regarding the capability of periphytic biofilms for simultaneous heavy metals removal and methane from aquatic ecosystems are scarce.

In the present study, a DNA-based stable isotope probing (DNA-SIP) technique was exploited to establish the correlation between the taxonomic identity of dynamic methanotrophs during heavy metal bioremediation and oxidization of methane in lake epipelon. The main objectives of the present study were: (1) to assess the heavy metals removal efficiency in association with increased methane oxidation capacity of epipelon; (2) to establish direct link between taxonomic identities of active methanotrophs and heavy metals bioremediation in epipelon; (3) to develop a novel strategy for mechanistic understanding of methanotroph-mediated feedback of microbial community structure for heavy metals bioremediation.

## 2. Materials and Methods

### 2.1. Epipelon Isolation and Growth conditions

Epipelon biofilm was obtained, following standard methods, from the sediments of Xuan Wu, Lake Nanjing by using a 5-cm diameter acrylic core. The supernatant water was heedfully removed with a pipette to prevent planktonic algal contamination [26]. For further analysis, samples were collected from the top (1 cm) of the extruded sediment. The average conditions of the sampling site were determined at the time of sampling. The collected biofilm was transported and grown in the laboratory as previously described [19,27]. The epipelon biofilm was grown in 5 L glass beakers covered with perforated Sigma-Aldrich Parafilm^®^. The temperature was set to 28 °C and the beakers were placed in a growth chamber under a specific time period regime for periphytons (16 h light + 8 h dark period). A continuous 8 h dark and 16 h light period was used because epipelon biofilms proved to be light sensitive [28]. pH was adjusted to 6.80 for promotion of microbial growth as previously reported [19,27]. After 90 days of incubation, the biofilm was suspended and 5 mL of suspended biofilm, along with 15 mL of modified WC (without Carbon Source) was added to each microcosm and transferred into a 120 mL serum bottle as one microcosm. All microcosms were kept in a growth chamber for 20 days to stabilize epipelon growth. After the stabilization of biofilm, microcosms were further employed for the methane oxidation and heavy metal removal studies.

### 2.2. DNA-SIP and Heavy Metals Bioremediation Analysis

Microcosms for methane gas oxidation were prepared with the following six treatments in triplicate including controls. (1) 6 ppm ^13^CH_4_, (2) 120,000 ppm ^13^CH_4_ (12%), (3) 120,000 ppm ^12^CH_4_, (4) 50 mg L^−1^ Cr^6+^ + 120,000 ppm ^13^CH_4_, (5) 50 mg L^−1^ Pb^2+^ + 120,000 ppm ^13^CH_4_, and (6) 50 mg L^−1^ Cd^2+^ + 120,000 ppm ^13^CH_4_. The headspace gas of microcosm comprised of 67% N_2_ and 21% O_2_ for incubation conditions under 120,000 ppm (12%) methane concentration, or of 78% N_2_ and 21% O_2_ for incubation under near atmospheric methane condition (6 ppm). The stock solutions of metal salts, i.e., K_2_Cr_2_O_7_, PbCl_2_ and CdCl_2_ (each 500 mg L^−1^) were formed using deionized water.

To study the effect of methane oxidation on heavy metal bioremediation, six treatments with high and near atmospheric methane dose along with N_2_ and O_2_ were carried out as described earlier using microcosms in the laboratory: (1) 50 mg L^−1^ Cr^6+^ + 120,000 ppm ^13^CH_4_, (2) 50 mg L^−1^ Pb^2+^ + 120,000 ppm ^13^CH_4_, (3) 50 mg L^−1^ Cd^2+^ + 120,000 ppm ^13^CH_4_, (4) 50 mg L^−1^ Cr^6+^ + 6 ppm ^13^CH_4_, (5) 50 mg L^−1^ Pb^2+^ + 6 ppm ^13^CH_4_ and (6) 50 mg L^−1^ Cd^2+^ + 6 ppm ^13^CH_4_.

For each microcosm, 20 mL epipelon mixture was incubated in serum bottles sealed with a butyl stopper, after replacing head space volume with respective gases [29]. The ^13^CH_4_ used in the study was > 99% ^13^C-atom pure (Cambridge Isotope Laboratories, Tewksbury, MA, USA). Gas chromatography 2010-pro (Shimadzu GC12-A, Kyoto, Japan) was used to measure the concentration of the headspace CH_4_ every 24 h. Microcosm incubation was considered complete when the methane concentration was ≤100 ppm, and the epipelon biofilm was collected from each microcosm right after the treatment and all the samples were stored at −20 °C for further analysis. In order to determine the relative abundance of ^13^C-atom after microcosm incubation, substantially, 1 g sample was vacuum freeze-dried and was analyzed by a Flash 2000 elemental analyzer coupled to a Delta V™ IRMS Advantage isotope ratio mass spectrometer (Thermo Scientific™, Waltham, MA, USA). For heavy metals determination, a few drops of concentrated nitric acid were added to avoid precipitation of metals as well as growth of biofilm [30]. The samples were stored at 4 °C for further analysis. The concentrations of Pb^2+^, Cd^2+^ and Cr^6+^ in the samples were evaluated by inductively coupled plasma mass spectrometry (ICP-MS).

### 2.3. Morphology and Activity of Microbes in Epipelon Biofilms

The morphology of the microorganism of epipelon biofilm was compared before and after treatment of 120,000 ppm ^13^CH_4_, by cell observation under SEM (EVO 18, Zeiss, Germany) and phase contrast microscopes (PCM) (Nikon Eclipse 135 Ti, Kyoto, Japan). The microbial activity for different substrates was assessed using the Biolog™ plate technique. Briefly, 2 g biofilm pellet was (centrifuged at 12,000 rpm and 25 °C) inoculated into plate wells [31] and was incubated at 25 °C for a week. The average well color development (AWCD) was monitored using a Biolog™ Microplate Reader ELx808 (590 nm) on a daily basis [27].

### 2.4. DNA Extraction and SIP Gradient Fractionation

DNA was extracted from 500 mg epipelon sample of each treatment using the FastDNA spin kit for soil (MP Biomedicals, Santa Ana, CA, USA), according to the manufacturer’s manual. The quality and quantity of epipelonic DNA was evaluated using a NanoDrop ND-1000 UV-visible light spectrophotometer (NanoDrop Technologies, Wilmington, DE, USA). For ^12^CH_4_ and ^13^CH_4_ fractions, ^13^C-labeled DNA was isolated from ^12^C DNA by using density gradient centrifugation of total DNA, as formerly described [32]. The fractionated DNA in the CsCl medium was precipitated by polyethylene glycol 6000 (PEG 6000) and diluted to 30 µL water for downstream analysis.

### 2.5. Real-Time Quantitative PCR of pmoA Genes

Methanotrophic biomarker *pmoA* genes were quantified by real-time quantitative PCR (qPCR) from total DNA to ascertain the abundances of Methane Oxidizing Bacteria (MOB) on a CFX96 Optical Real-Time Detection System (Bio-Rad, Irvine, CA, USA). Fractionated DNA gradients of no. 2–14 were also used for the qPCR analysis of *pmoA* genes to assess the competence of ^13^C amalgamation into the genomic DNA of MOB communities. The primer pair and thermal cycling condition are given in details in the Supplementary Material (Table S1). PCR reaction mixtures and the standards were prepared and utilized as described previously [29]. Amplification efficiency ranged from 92% to 99%, with R^2^ values of 0.996 to 0.999.

### 2.6. MiSeq Sequencing of 16S rRNA Genes

Amplicon sequencing of 16S rRNA genes was conducted for the total DNA extracts, as well as DNA from the heavy CsCl fractions (buoyant density around 1.735 g mL^−1^) recovered from ^13^CH_4_-amended microcosms [33], using universal bacterial primer pair 515F/907R. Fractions with the identical buoyant density from ^12^CH_4_ microcosms was also sequenced to provide background details. Low-quality sequences were eliminated from generated reads by applying key-quality control steps [34] using QIIME pipeline [35]. A total of 696,738 sequences from 16S rRNA genes were obtained with quality score > 20, without mismatched primers and ambiguous bases (Table S2) and RDP MultiClassifier was used to obtain taxonomic assignments [36]. The relative abundance of type I methanotrophs was obtained as an aggregate of sequences associated to *Methylobacter*, *Methylomonas*, *Methylomicrobium*, *Methylosoma* and *Methylococcus* and type II methanotrophs included *Methylosinus* and *Methylocystis* sequences. All MOB-affiliated 16S rRNA gene sequences were collected and corporated into OTU at 97% identity threshold [37]. For ^13^C-labeled 16S rRNA genes, representative sequences of dominant OTUs (containing ≥ 2% of MOB-like 16S rRNA gene sequences in at least one of the selected samples) were applied for the phylogenetic tree analysis using the neighbor-joining method in latest MEGA-X version with bootstrapping value of 1000 replicates in all treatments [38].

### 2.7. MiSeq Sequencing of pmoA Genes

Amplicon sequencing of *pmoA* genes was conducted on DNA from heavy fractions of ^13^CH_4_-amended microcosms using A189f/mb661r, using a similar strategy as that described previously (Cai et al., 2016). The generated *pmoA* gene reads were applied to bioinformatic processing key steps as previously described [33,39]. Molecular analysis was based on a total of 244,839 high-quality sequences (Table S3). For the major *pmoA* OTUs (containing ≥ 3% of *pmoA* gene sequences in at least one of the samples), an emblematic sequence was utilized for the phylogenetic analysis by associating with previously reported sequences from GenBank.

### 2.8. Statistical and Network Analysis

The Vegan package in R 3.4.3 (https://www.reproject.org/) was employed to compute the α-diversity indices (OTU richness, Chao1, Simpson index, Evenness index, and Shannon index) of epipelon biofilm samples.

Network visualization and modular analyses of 16S rRNA sequence results were obtained using Gephi (http://gephi.github.io/). The topological properties of the networks, including betweenness centrality, average degree, average path length, and modularity were also computed using Gephi for all treatments. The size of each node is directly proportional to the relative abundance of the bacterial community (log (*n*+1)).

The functional capacity of the epipelon biofilm samples was assessed by using the Phylogenetic Investigation of Communities by Reconstruction of Unobserved States (PICRUSt) version 1.0.0 [40]. A similarity threshold of 97% of all OTUs was used in QIIME for the prediction of PICRUSt and the Green genes database (13_5_release) was employed as a clustering reference while metagenomic functional profiles were prognosticated using the *predict_metagenomes.py* script.

## 3. Results and Discussion

### 3.1. Physiochemistry, Cell Morphology and Activity of Epipelon Biofilm

**Figure 1:**
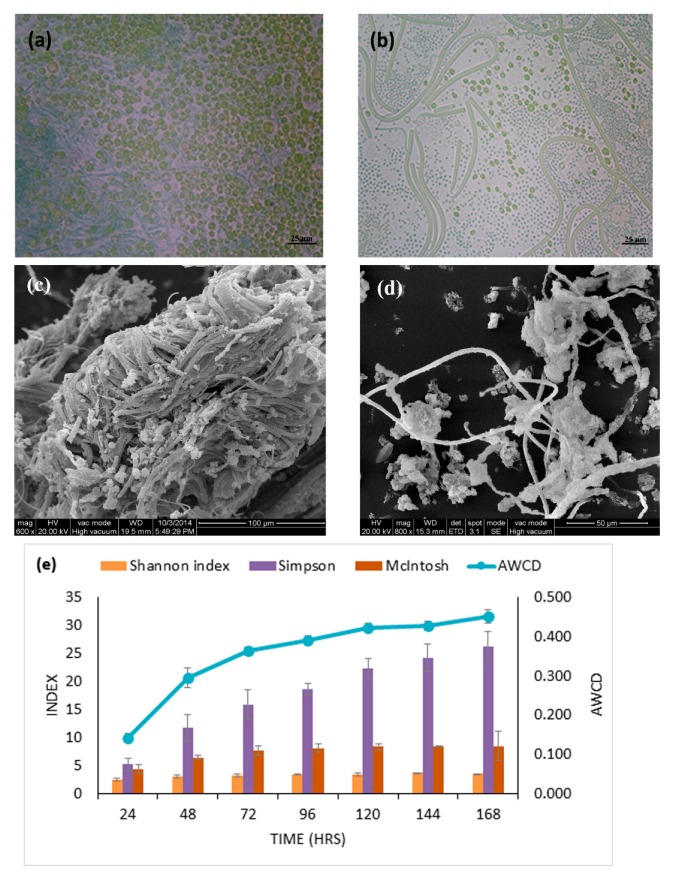
Phase Contrast Microscopy (PCM) and Scanning Electron microscopy (SEM) were performed to differentiate microbial community change (**a**) and (**b**) community composition of epipelon observed under PCM before and after 12% methane treatment, respectively. (**c**) and (**d**) structure of periphytic biofilm observed under SEM before and after 12% methane treatment, respectively. (**e**) The average well color development (AWCD) of the Biolog EcoPlates at 590 nm for periphytic biofilm and diversity indices (E) for species diversity.

The physicochemical properties at the sampling site of epipelon biofilm were: ammonia 0.66 mg L^−1^, nitrate 0.78 mg L^−1^, total phosphorus (TP) 0.23 mg L^−1^, total nitrogen (TN) 2.12 mg L^−1^ and pH 7.78. Epipelon are an integration of a wide variety of microorganisms, including algae, bacteria and protozoa, that collaboratively live in or on fine-grained substrata or sand. However, these biofilms under ambient methane concentration were predominantly composed of cyanobacteria and phytoplankton (Figure 1a). Moreover, after incubation under 120,000 ppm methane, bacterial species outnumbered cyanobacteria and phytoplankton as shown by PCM (Figure 1b). SEM showed that the structure of epipelon biofilm was intertwined bundles, with voids of algae, and diatoms, while bacteria attached to these were evident in these structures. The spaces and tunnels found within epipelonic biofilms are likely to provide physical space for bacterial attachment and metals sorption sites and are clearly visible in the SEM (Figure 1c,d) [41].

The capability of different types of periphytic biofilms in the bioremediation and biosorption of different contaminants from physiochemically contrasting lakes has been intensively reported [42,43]. However, no studies have yet been reported about enhanced heavy metals removal by epipelon under a high methane regime which could likely represent naturally occurring conditions in a wetland.

In Biolog^®^ experiments, the Average Well Color Development (AWCD) is an indicator of the degree of activity of epipelon biofilm. The darker color of the wells represents the higher activity of the microorganisms as confirmed from the slope of the graph (Figure 1e). The diversity indices showed higher community abundance, higher functional activity of epipelon, uniformity and species eveneness of the microbial community (Figure 1e), e.g., after incubation of Biolog^®^ for 7 days, the Shannon index was found to be 3.6. This result indicates higher microbial diversity and species evenness in epipelon when compared to the control.

### 3.2. Effect of Methane Oxidation on Heavy Metal Bioremediation

**Figure 2:**
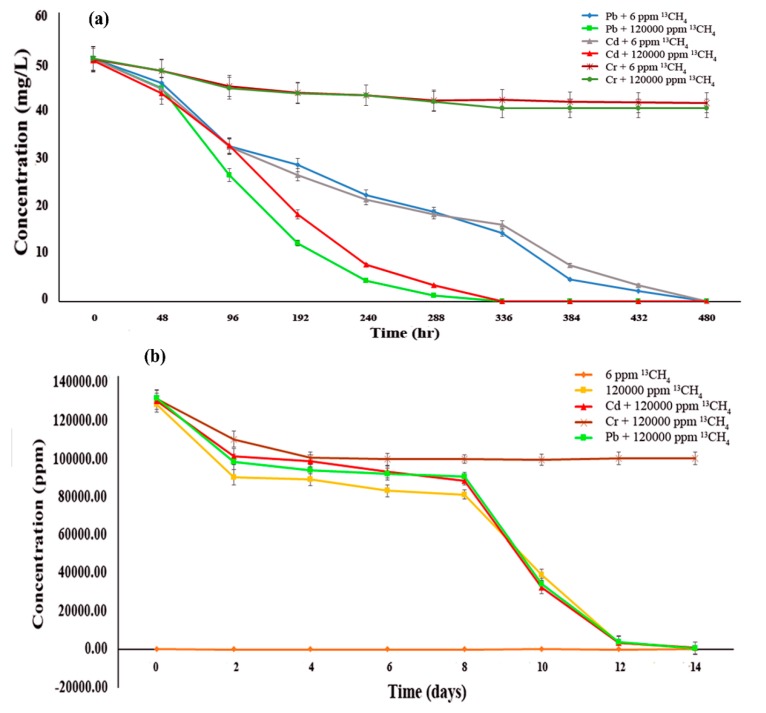
Methane oxidation and heavy metals bioremediation potential of epipelon (**a**) the ability of epipelonic biofilm to remove heavy metals at different time intervals coupled with methane oxidation (**b**) Methane oxidation at different time intervals under heavy metals effect.

Epipelon was found to be highly active in removing Pb^2+^ and Cd^2+^ under 12% (120,000 ppm) methane, with 100% removal efficiency within 336 h (Figure 2a), although only 20% of Cr^6+^ was removed within same interval of time. It should also be noted that the low removal efficiency of Cr^6+^ could not entirely reflect the operating conditions in natural environments because Cr^6+^ is toxic in laboratory scale experiments without continuous recirculation, as previously suggested [44]. Meanwhile, hexavalent chromium is very toxic due to its high water-solubility and strong oxidizing ability. Chromium (VI) posed a toxicity risk to periphytic biofilm via inhibition of its photosynthetic activity and carbon utilization function [45], thus inducing destruction of the biofilm, which ultimately results in releasing Cr^6+^ back into the environment, making it more toxic. For instance, hexavalent chromium removal efficiency decreased from 90% to 50% for *E. crassipes* when compared to trivalent chromium [46]. A free water surface wetland made from sediment was found to be only capable of 7% chromium removal [47]. Furthermore, the isotope fractionation factor (ε) of Cr^6+^ was 2.62 ± 0.20% after 509 days of continuous incubation with methane based on theoretical estimation using the Rayleigh distillation mode [48] during slow bioremediation of Cr^6+^ by methanotrophs. In one similar periphytic study, Cd(II) was more secluded in comparison to Cr(VI), while field validation of this observation remained an intriguing issue for further research [49].

It was further observed that under ambient methane concentration (6 ppm), epipelon completely removed amended Pb^2**+**^ or Cd^2**+**^ (both at 50 mg L^−1^) within 480 hrs, but the incubation under high methane concentration (120,000 ppm) greatly enhanced the metal removal efficiencies, which took only 336 hrs to complete the process (Figure 2a). This might be attributed to saturation effect of the active sites on the surface of the epipelonic biofilms. Comparatively, limited sensitivity of methane oxidation was found after adding Pb(II), which might have been due to soil water content as the primary key force for Pb(II) removal [50].

The patterns of metal tolerance, as shown by methane oxidizers, are comparatively distinct to chloride salts of Cd(II), Cr(III), Hg(II), Cu(II)and Zn(II) [51]. Another interesting aspect of the novel isolates of methanotrophs was their resistance to various types of pollutions (heavy metals, arsenate or organic) and were considered to be real ‘super-bugs’ due to their capability to withstand remarkably high concentration of various pollutants [52]. The abundance of extracellular polymeric substances, plays a key role in the higher removal efficacies of periphytic biofilms [21] as well as in-tank microbial activity [53].

The potential implication of methane monooxygenases, for bioremediation of different contaminants including heavy metals, is accentuated by their broad substrate profiles [54]. Hence, epipelonic biofilms have proven to be considerably effectual towards the removal of heavy metals within a shorter time period under high methane doses compared to previous studies, thus providing a reliable source for the removal of heavy metals from aquatic environments.

### 3.3. Potential of Epipelon for Methane Oxidation and ^13^C Assimilation

All treatments were found to exhibit strong methane oxidation activity and consumed more than 99% of amended methane except for 6 ppm ^13^CH_4_ treatment, which shows no significant methane oxidation, and 50 mg L^−1^ Cr^6+^ + 120,000 ppm ^13^CH_4_ only utilized 23.94% of available methane (Figure 2b). No significant difference in methane oxidation rate was observed between ^12^CH_4_ and ^13^CH_4_ treatment in microcosms with high concentration.

The assimilation of methane derived carbon obtained by methane oxidation was increased in organic carbon in high dose (12%) methane treatments. The percentage of ^13^C atom was significantly increased from 1.08% ^13^C atom in the near-atmospheric dose ^13^C methane (6 ppm) treatment and in high-dose ^12^C methane treatments to 16.24%, 12.14% and 13.78% ^13^C atoms in 120,000 ppm ^13^CH_4_, 50 mgL^−1^ Cd^2+^ + 120,000 ppm ^13^CH_4_ and 50 mgL^−1^ Pb^2+^ + 120,000 ppm ^13^CH_4_ treatments, respectively (Figure S1). These results confirm the potential of epipelonic biofilms for the assimilation of methane in higher amounts, thus confirming their potential towards effective methane oxidation in aquatic environments.

### 3.4. Methanotrophic Community Abundance in Response to Heavy Metals

**Figure 3:**
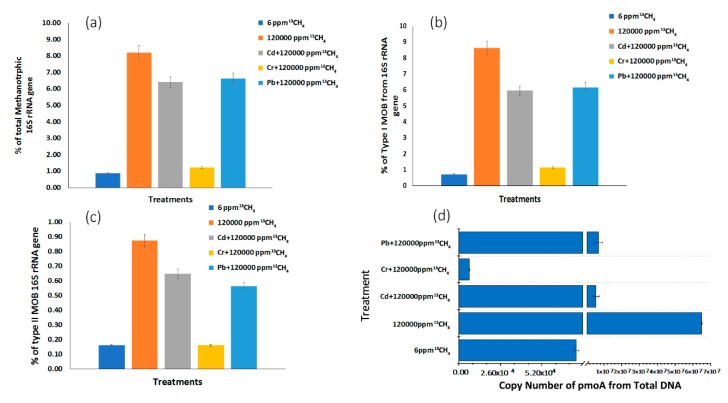
Illumina sequencing was carried out at the whole epipelon microbial community level in microcosms targeting total methanotrophs from total 16S rRNA genes (**a**), and the relative abundances of type I (**b**), type II (**c**), The *pmoA* gene copy numbers were assessed using real-time quantitative PCR (**d**).

The abundance of the total microbiome and methanotrophs in epipelon was evaluated by real-time quantitative PCR of 16S rRNA and *pmoA* genes, respectively. Heavy metals significantly inhibit the abundance of total microbial communities (Figure 3d). Compared to control treatment (120,000 ppm ^13^CH_4_), the number of 16S rRNA genes was 37.94% and 26.49% lower in microcosm amended with Cd^2^ and Pb^2+^, respectively (Figure 3d). In previous studies, single cultures of methanotrophic bacteria were verified for bioremediation of heavy metals such as chromium [17,52,55]. Although the favorable conditions were revealed for a single type of strain, it is not a cost-effective and sustainable solution for heavy metal removal on a large scale. In the present study, the presence of Cr^6+^ in 50 mg L^−1^ Cr^6+^ + 120,000 ppm ^13^CH_4_ treatment resulted in a lower abundance (6.97 less folds) of methanotrophs due to Cr^6+^ toxicity as compared to 120,000 ppm ^13^CH_4_ whereas the treatments with lead (1.20 less folds) and cadmium (1.57 less folds) did not show much effect on the methanotrophic community in biofilms.

On the other hand, 120,000 ppm ^13^CH_4_ got 884, 12.13 and 9.46 fold higher MOB than 6 ppm ^13^CH_4_, 50 mg L^−1^ Cd^2+^+ 120,000 ppm ^13^CH_4,_ 50 mg L^−1^ Pb^2+^+ 120,000 ppm ^13^CH_4_, respectively (Figure 3d).

According to high-throughput sequencing of 16S rRNA genes, high methane treatments (120,000 ppm ^13^CH_4,_ 50 mg L^−1^ Cd^2+^ + 120,000 ppm ^13^CH_4_ and 50 mg L^−1^ Pb^2+^+ 120,000 ppm ^13^CH_4_ ) got 9.7, 7.35, 7.69 more folds of MOB genes, respectively, compared to near-atmospheric methane treatment (6 ppm ^13^CH_4_) (Figure 3a). Chromium-augmented treatment showed only 1.41 more folds of MOB genes than near atmospheric methane treatment. This might be due to toxic nature of chromium that suppressed the abundance of MOB genes in the following treatments.

The abundance of type I and type II MOB in the whole microbial community was determined using high-throughput sequencing of 16S rRNA genes data. Type I methanotrophs copy numbers were increased by factors of 12.16, 8.39, 8.69 fold in the high methane treatments (120,000 ppm ^13^CH_4,_ 50 mg L^−1^ Cd^2+^ + 120,000 ppm ^13^CH_4_ and 50 mg L^−1^ Pb^2+^ + 120,000 ppm ^13^CH_4)_ compared to near-atmospheric methane treatments (6 ppm) (Figure 3b). Type II MOB was also stimulated by 5.43, 4.02, 3.50 and 1.01 folds in 120,000 ppm ^13^CH_4,_ 50 mg L^−1^ Cd^2+^ + 120,000 ppm ^13^CH_4,_ 50 mg L^−1^ Pb^2+^ + 120,000 ppm ^13^CH_4_ and 50 mg L^−1^ Cr + 120,000 ppm ^13^CH_4_, respectively, as compared to 6 ppm ^13^CH4 (Figure 3c).

The chromium-augmented treatment showed only a 1.61-fold increase for the high methane as compared to near atmospheric methane treatment (6 ppm). The sequence analysis of the microbial community from lake Biwa, Japan [3,56], stratified eutrophic Swiss lakes [57] and Lacamas lake in Washington, USA [58] showed Type I methanotroph dominances, similarly to our findings in this study.

### 3.5. SIP for Identification of Active Methanotrophs

**Figure 4:**
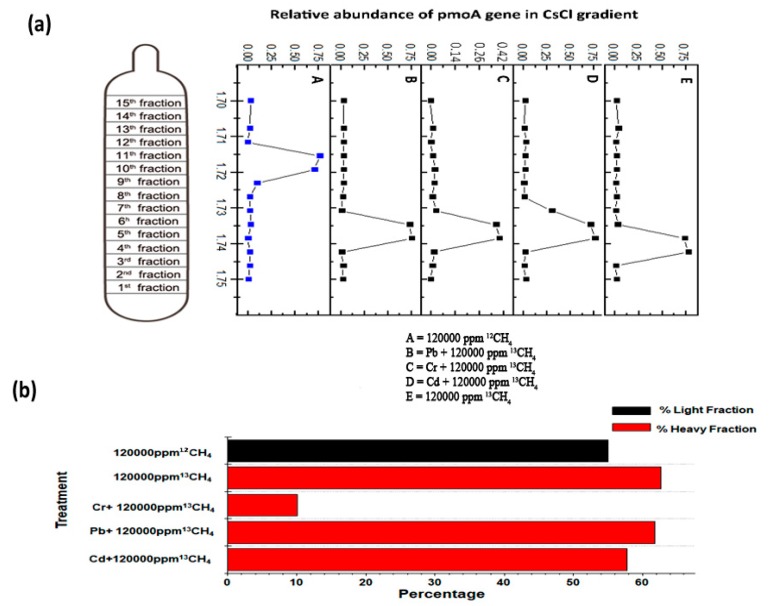
The enrichment of 13Clabeled methanotrophs on the basis of qPCR of *pmoA* gene (**a**). The relative *pmoA* gene abundance was the proportion of MOB like gene copy numbers in each fraction to the total abundance through the gradient in a treatment (**b**). The red columns represent the percentage of MOB affiliated reads in total 16S rRNA genes in the heavy fractions (buoyant density 1.745 g mL^−1^) while the black column represents light fraction (buoyant density 1.72 g mL^−1^).

The heavy fraction resolved by isopycnic centrifugation of the DNA was employed for qPCR of *pmoA* genes and clearly indicates active cell propagation during the consumption of ^13^C-methane in all the high methane dose treatments, except for the Cr^6+^ amended (10.05%) treatment and ambient ^13^CH_4_ (6 ppm) treatment. A peak shift of relative *pmoA* gene abundances (Figure 4a) towards heavy fractions (buoyant density around 1.745 g mL^−1^) was very evident in all high-dose ^13^CH_4_ treatments, while the ^12^CH_4_ *pmoA* gene abundances peaked only in the light fractions (buoyant density around 1.72 g mL^−1^) [29]. In ^13^C-labeled heavy DNA fractions (buoyant density around 1.745 g mL^−1^), the number of *pmoA* genes was significantly increased to 57.73%, 61.79% and 62.62% in 120,000 ppm ^13^CH_4_, 50 mg L^−1^ Cd^2+^ + 120,000 ppm ^13^CH_4_ and 50 mg L^−1^ Pb^2+^ + 120,000 ppm ^13^CH_4_, respectively (Figure 4b) as compared to the non-significant ^13^CH_4_ (6 ppm) treatment.

### 3.6. Microbial Community Response to Heavy Metals Bioremediation and Methane Oxidation

The structures and abundances of microbial communities were drastically changed due to the selection of distinct phylotypes in the different heavy metal treatments, along with methane oxidation (Figure S2). The taxonomic analysis of heavy metal resistant genes revealed that the most common bacterial phyla in the treatments was *Proteobacteria* followed by *Actinobacteria* [59] which is in consistent with our study. Microbial community composition and interaction was analyzed by high throughput sequencing to determine the impact of metals and methane oxidation on microbial community structure. The taxonomic classification of OTUs at different levels produced 28 phyla, 92 classes, 178 orders, 334 families and 751 genera. Approximately 28 different phyla were found to be present in sequencing of the epipelonic biofilm samples, among these Proteobacteria, Cyanobacteria, Saccharibacteria and Gemmatimonadetes were the most abundant phyla in all treatments (Figure S3). The phylum Proteobacteria was found to be the most dominant in 120,000 ppm ^13^CH_4_. In comparison to other treatments, it outnumbered 50 mg L^−1^ Pb^2+^ +120,000 ppm ^13^CH_4_, 50 mg L^−1^ Cd^2+^ + 120,000 ppm ^13^CH_4_ and 6 ppm ^13^CH_4_ by 13.66%, 17.23% and 22%, respectively. Alpha and gamma Proteobacteria have recently been shown to be effectual for the bioremediation of different heavy metals such as cadmium in an iron reducing bacterial immobilization system [60].

**Figure 5:**
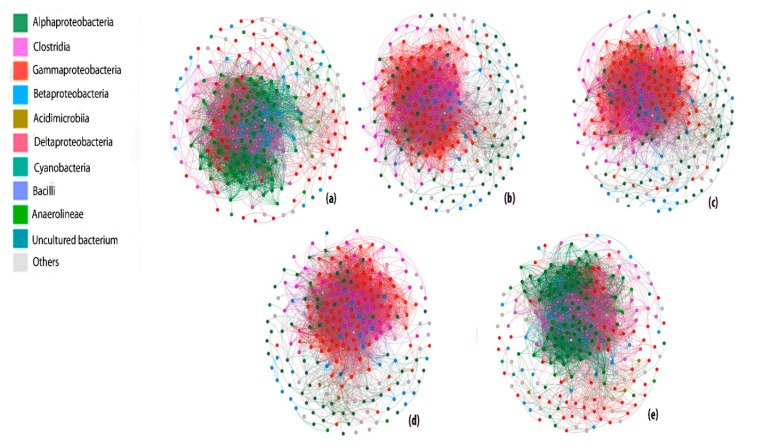
The co-occurrence network analysis of bacterial communities in the order (**a**) 6 ppm ^13^CH_4_ (**b**) 120,000 ppm ^13^CH_4_ (**c**) 50 mg L^−1^ Pb^2+^ + 120,000 ppm ^13^CH_4_ (**d**) 50 mg L^−1^ Cd^2+^ + 120,000 ppm ^13^CH_4_ (**e**) 50 mg L^−^^1^ Cr^6+^ + 120,000 ppm ^13^CH_4_. The network represents strong (Spearman’s r > 0.8) and significant (*p* value < 0.01) correlation. Each node represents a unique sequence in the whole data set, colored nodes (excluding light gray) depicts the ten major groups while light gray represents all other groups.

On the basis of Spearman correlation analysis, a co-occurrence network of different co-existing communities 120,000 ppm ^13^CH_4_ comprised of 287 nodes, 5128 edges, average degree 1.87, highest graph density 0.182, highest modularity 0.193 and average network distance 2.58 was generated, indicating strong microbial interactions (Figure 5). Ambient 6 ppm ^13^CH_4_ and 50 mg L^−1^ Cr^6+^ + 120,000 ppm ^13^CH_4_ treatments were dominated by alpha-Proteobacteria but the scenario was changed to gamma Proteobacteria in high methane dose treatments as represented by colored dots in co-occurrence network. Based on the level of interactions from all the calculated modules, we can arrange treatments in the following decreasing order of interaction: 120,000 ppm ^13^CH_4_ > 50 mg L^−1^ Cd^2+^ + 120,000 ppm ^13^CH_4_ > 50 mg L^−1^ Pb^2+^ + 120,000 ppm ^13^CH_4_ > 6 ppm ^13^CH_4_ > 50 mg L^−1^ Cr^6+^ + 120,000 ppm ^13^CH_4_ (Table S4). The modularity of all the networks differs from each other, demonstrating the verity that the modularity of different networks with different doses of methane or metals or contaminants changed [61]. However, the strong correlation between nodes illustrates that the microbe–microbe interaction stayed strong after the intrusion of external sources or by the bioadsorption of heavy metals or methane oxidation.

Phylogenetic Investigation of Communities by Reconstruction of Unobserved States (PICRUSt) analysis represented functional attributes of all treatments, 120,000 ppm ^13^CH_4_, 50 mg L^−1^ Cd^2+^ + 120,000 ppm ^13^CH_4_ and 50 mg L^−1^ Pb^2+^ + 120,000 ppm ^13^CH_4_ was dominated by methanotrophy (Figure S4) followed by chemohetrotrophy, aerobic chemohetrotrophy, and phototrophy. Pheatmap confirms the presence of genes engaged in numerous processes such as cellular processes, environmental information processing, metabolism, and genetic information processing [62].

We further investigated the presence of dominant species at phylum, class, order, and family level by linear discriminant analysis effect size pipeline (LEfSe) analysis. Proteobacteria dominated among all treatments while the 50 mg L^−1^ Cr^6+^ + 120,000 ppm ^13^CH_4_ community was dominated by Cyanobacteria and Acidobacteria (Figure S5). The microbial community in treatments, 50 mg L^−1^ Cr^6+^ + 120,000 ppm ^13^CH_4_ was predominately composed of Verrucomicrobia, *Methylomonas* and Fimbriimonadales.

### 3.7. Methanotrophic Community Composition

**Figure 6:**
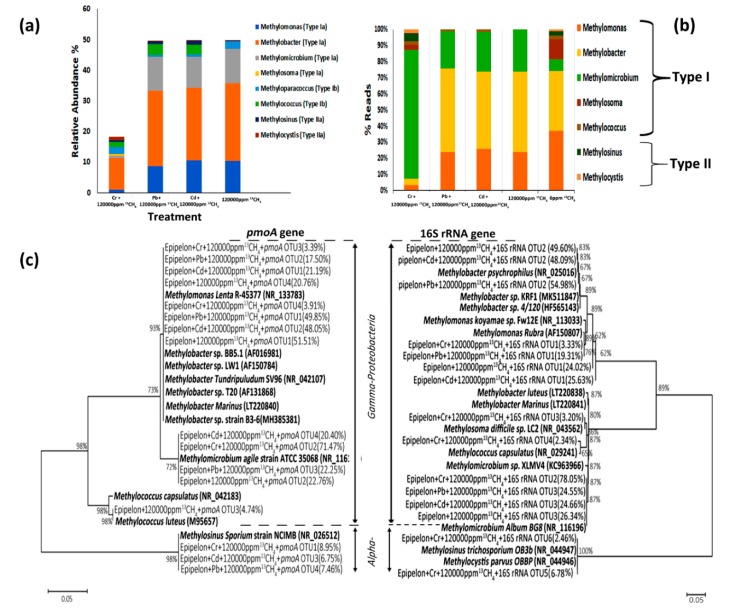
Methanotrophic community compositions of 13C-labeled MOB based on phylogenetic tree analysis of 16S rRNA and *pmoA* genes. (**a**) Percentage of type Ia, Ib, II and IIa methanotrophs were computed from the 13C-labeled genes. (**b**) Percentages of type I and II MOB were determined from16S rRNA high throughput sequencing of the 13C-labeled genes (**c**) Neighbor-joining phylogenetic tree depicts the phylogenetic relationship of dominant OTU sequences with the known MOB sequences from GenBank. The percentages illustrate the relative sequence abundance of each OTU to the total MOB-affiliated 16S rRNA or *pmoA* genes. Bootstrap values higher than 60% are pointed at the branch nodes.

Taxonomic analysis of ^13^C-labeled methanotrophic 16S rRNA genes further demonstrated distinct proportions of active type I and II methane oxidizers in all treatments (Figure 6b). Particularly, *Methylobacter* a type I methanotroph was 51.81%, 48.09%, 49.59% abundant in 50 mg L^−1^ Pb^2+^ + 120,000 ppm ^13^CH_4_, 50 mg L^−1^ Cd^2+^ + 120,000 ppm ^13^CH_4_, 120,000 ppm ^13^CH_4_ treatments, respectively (Figure 6a). On the contrary, *Methylomicrobium* was 80.04% abundant in 50 mg L^−1^ Cr^6+^ + 120,000 ppm ^13^CH_4_. These results were further confirmed by neighbor-joining tree-clustering around *Methylobacter* spp. (Figure 6c).

Thus, it was demonstrated that the abundance of *Methylobacter* sp. by the ^13^C-labeled *pmoA* genes. Type I outnumbered Type II with percentages of 90.65%, 90.91% and 99.80% of total methanotrophs in 50 mg L^−1^ Pb^2+^ + 120,000 ppm ^13^CH_4_, 50 mg L^−1^ Cd^2+^ + 120,000 ppm ^13^CH_4_ and 120,000 ppm ^13^CH_4_, respectively. Type II MOBs, *Methylosinus* and *Methylocystis,* were stimulated by high methane dose and metal contamination in treatments 50 mg L^−1^ Pb^2+^ + 120,000 ppm ^13^CH_4_ and 50 mg L^−1^ Cd^2+^ + 120,000 ppm ^13^CH_4_ by 9.14% and 8.8% as compared to 120,000 ppm ^13^CH_4_.

*Methylobacter* spp. and *Rhodoferax* spp. were found to be the most dominant species in heavy metals contaminated landfill [63]. Zolgharnein, et al. [64] have evaluated the role of *Delfetia* and *Methylobacter* spp. in the removal of heavy metals such as lead, zinc, copper and cadmium, for the first time. They reported that in the absence of Cu(II) or Cu(I), methanobactin from *Methylosinus trichosporium* OB3b will bind Ag(I), Co(II), Au(III), Cd(II), Hg(II), Fe(III), Ni(II), Mn(II), Pb(II), Zn(II), or U(VI) which represents the ability of *Methylosinus* to bioremediate heavy metals [55]. Similarly, *Methylococcus capsulatus* has been found to detoxify lead and zinc at low pH [65] and chromium [17]. The type II methanotrophic strains identified in this study have been widely utilized for the bioremediation of heavy metals [16,66].

These results imply that many methanotrophs, including *Methylobacter*, *Methylococcus, Methylosinus, Methylocystis* and *Methylmicrobium* have an unacknowledged metabolic potential and multifaceted capabilities for bio-remediating heavy metals. Higher type I methanotrophic activities were confirmed in all treatments, which might be due to the higher impact of intrinsic biotic and abiotic characteristics of Xuan Wu Lake.

## 4. Conclusions

In the present study, the potential capability of naturally occurring epipelon biofilms for the simultaneous removal of heavy metals along with methane oxidation was assessed. The results show that high methane dose (12%), has a substantial effect on heavy metals (Pb, Cd, Cr) removal. Epipelon exhibits a strong methane oxidation potential and 100% removal efficiency was achieved for Pb^2+^ and Cd^2+^ within 336 h, whereas Cr^6+^ was only removed by as little as 20% even after 480 h. Under high methane concentration (120,000 ppmv), bioremediation capability of epipelon is enhanced in terms of time. MOBs along with other co-existing microbes are dominated by type I after treating with 120,000 ppm (12%) headspace methane. The addition of Cr^6+^ has a negative impact on the epipelon community structure and inhibits methanotrophs along with other species. Heavy metals cause reduced abundance of certain microbes as compared to control treatments with 12% methane in the absence of metals. It is concluded from the present study that epipelon have strong potential to be used as an environmentally friendly technology which can be used for the simultaneous removal of different types of pollutants in aqueous and atmospheric environments.

## Supplementary Materials

The following are available online at https://www.mdpi.com/2076-2607/8/4/505/s1, Table S1. Primers and PCR conditions used in this study. Table S2. Summary of MiSeq amplicon sequencing of 16S rRNA genes from total DNA sample. Table S3. Summary of Illumina MiSeq amplicon sequencing of *pmoA* genes from total DNA. Table S4 Topological properties of the co-occurrence networks of lake microbial communities. Figure S1. Percentage of ^13^C atoms abundance accumulated by methanotrophs in Epipelon. Figure S2. The changes in community composition of microbes following microcosm incubation with respective methane doses based on high throughput sequencing of 16S rRNA genes at class level. Figure S3. The changes in community composition of microbes following microcosm incubation with respective methane doses based on high throughput sequencing of 16S rRNA genes at phylum level. Figure S4. Functional pheat map of Epipelon under different methane and metals doses. Figure S5. linear discriminant analysis effect size pipeline (LEfSe) analysis for dominant phyla, class and species.

## References

[1] Duren R.M., Thorpe A.K., Foster K.T., Rafiq T., Hopkins F.M., Yadav V., Bue B.D., Thompson D.R., Conley S., Colombi N.K. (2019). California’s methane super-emitters. Nature.

[2] Rai P.K., Lee S.S., Zhang M., Tsang Y.F., Kim K.-H. (2019). Heavy metals in food crops: Health risks, fate, mechanisms, and management. Environ. Int..

[3] Iguchi H., Umeda R., Taga H., Oyama T., Yurimoto H., Sakai Y. (2019). Community composition and methane oxidation activity of methanotrophs associated with duckweeds in a fresh water lake. J. Biosci. Bioeng..

[4] Landrigan P.J., Fuller R., Acosta N.J.R., Adeyi O., Arnold R., Basu N., Baldé A.B., Bertollini R., Bose-O’Reilly S., Boufford J.I. (2018). The Lancet Commission on pollution and health. Lancet.

[5] Laxmi V., Kaushik G., Saxena G., Bharagava R.N. (2020). Toxicity of Hexavalent Chromium in Environment, Health Threats, and Its Bioremediation and Detoxification from Tannery Wastewater for Environmental Safety. Bioremediation of Industrial Waste for Environmental Safety: Volume I: Industrial Waste and Its Management.

[6] Fu Z., Xi S. (2020). The effects of heavy metals on human metabolism. Toxicol. Mech. Methods.

[7] Bhakta J., Munekage Y., Ohnishi K., Jana B. (2012). Isolation and identification of cadmium-and lead-resistant lactic acid bacteria for application as metal removing probiotic. Int. J. Environ. Sci.Technol..

[8] Doggaz A., Attoura A., Mostefa M.L.P., Côme K., Tlili M., Lapicque F. (2019). Removal of heavy metals by electrocoagulation from hydrogenocarbonate-containing waters: Compared cases of divalent iron and zinc cations. J. Water Process Eng..

[9] Abdullah N., Yusof N., Lau W., Jaafar J., Ismail A. (2019). Recent trends of heavy metal removal from water/wastewater by membrane technologies. J. Ind. Eng. Chem..

[10] Soto J., Ortiz J., Herrera H., Fuentes A., Almonacid L., Charles T.C., Arriagada C. (2019). Enhanced Arsenic Tolerance in Triticum aestivum Inoculated with Arsenic-Resistant and Plant Growth Promoter Microorganisms from a Heavy Metal-Polluted Soil. Microorganisms.

[11] Wang Y., Zhong B., Shafi M., Ma J., Guo J., Wu J., Ye Z., Liu D., Jin H. (2019). Effects of biochar on growth, and heavy metals accumulation of moso bamboo (Phyllostachy pubescens), soil physical properties, and heavy metals solubility in soil. Chemosphere.

[12] Khatiwada B., Hasan M.T., Sun A., Kamath K.S., Mirzaei M., Sunna A., Nevalainen H. (2020). Probing the Role of the Chloroplasts in Heavy Metal Tolerance and Accumulation in Euglena gracilis. Microorganisms.

[13] Hazen T. (2018). Consequences of Microbial Interactions with Hydrocarbons, Oils, and Lipids: Biodegradation and Bioremediation.

[14] Chan S.I., Lee S.J., Lee E.Y. (2019). The Biochemistry of Methane Monooxygenases. In Methanotrophs: Microbiology Fundamentals and Biotechnological Applications.

[15] Lai C.-Y., Zhong L., Zhang Y., Chen J.-X., Wen L.-L., Shi L.-D., Sun Y.-P., Ma F., Rittmann B.E., Zhou C. (2016). Bioreduction of Chromate in a Methane-Based Membrane Biofilm Reactor. Environ. Sci. Technol..

[16] Shi L.-D., Chen Y.-S., Du J.-J., Hu Y.-Q., Shapleigh J.P., Zhao H.-P. (2019). Metagenomic Evidence for a Methylocystis Species Capable of Bioremediation of Diverse Heavy Metals. Front. Microbiol..

[17] Al Hasin A., Gurman S.J., Murphy L.M., Perry A., Smith T.J., Gardiner P.H.E. (2010). Remediation of Chromium(VI) by a Methane-Oxidizing Bacterium. Environ. Sci. Technol..

[18] Tavares D.A., Lambrecht R.W., de Almeida Castilho M.C., Henry R., Ferragut C. (2019). Epipelon responses to N and P enrichment and the relationships with phytoplankton and zooplankton in a mesotrophic reservoir. Aquat. Ecol..

[19] Shabbir S., Faheem M., Ali N., Kerr P.G., Wu Y. (2017). Evaluating role of immobilized periphyton in bioremediation of azo dye amaranth. Bioresour. Technol..

[20] Yang J., Liu J., Wu C., Kerr P.G., Wong P.-K., Wu Y. (2016). Bioremediation of agricultural solid waste leachates with diverse species of Cu (II) and Cd (II) by periphyton. Bioresour. Technol..

[21] Liu J., Wang F., Wu W., Wan J., Yang J., Xiang S., Wu Y. (2018). Biosorption of high-concentration Cu (II) by periphytic biofilms and the development of a fiber periphyton bioreactor (FPBR). Bioresour. Technol..

[22] Tang C., Sun P., Yang J., Huang Y., Wu Y. (2019). Kinetics simulation of Cu and Cd removal and the microbial community adaptation in a periphytic biofilm reactor. Bioresour. Technol..

[23] Lu H., Dong Y., Feng Y., Bai Y., Tang X., Li Y., Yang L., Liu J. (2020). Paddy periphyton reduced cadmium accumulation in rice (Oryza sativa) by removing and immobilizing cadmium from the water–soil interface. Environ. Pollut..

[24] Li Y., Song S., Xia L., Yin H., García Meza J.V., Ju W. (2019). Enhanced Pb(II) removal by algal-based biosorbent cultivated in high-phosphorus cultures. Chem. Eng. J..

[25] Jasrotia P., Ogram A. (2008). Diversity of nifH Genotypes in Floating Periphyton Mats Along a Nutrient Gradient in the Florida Everglades. Curr. Microbiol..

[26] Cano M.G., Casco M.A., Solari L.C., Mac Donagh M.E., Gabellone N.A., Claps M.C. (2008). Implications of rapid changes in chlorophyll-a of plankton, epipelon, and epiphyton in a Pampean shallow lake: An interpretation in terms of a conceptual model. Hydrobiologia.

[27] Shabbir S., Faheem M., Wu Y. (2018). Decolorization of high concentration crystal violet by periphyton bioreactors and potential of effluent reuse for agricultural purposes. J. Clean. Prod..

[28] Pniewski F.F., Richard P., Latała A., Blanchard G. (2018). Long- and short-term photoacclimation in epipsammon from non-tidal coastal shallows compared to epipelon from intertidal mudflat. J. Sea Res..

[29] Sultana N., Zhao J., Zheng Y., Cai Y., Faheem M., Peng X., Wang W., Jia Z. (2019). Stable isotope probing of active methane oxidizers in rice field soils from cold regions. Biol. Fertil. Soils.

[30] Ziadat A.H., Jiries A., Alojail I. Accumulation of Heavy Metals on Soil Irrigated with Treated Wastewater at Al al-Bayt University-Jordan. Proceedings of the 2019 Advances in Science and Engineering Technology International Conferences (ASET).

[31] Shabbir S., Faheem M., Ali N., Kerr P.G., Wang L.-F., Kuppusamy S., Li Y. (2020). Periphytic biofilm: An innovative approach for biodegradation of microplastics. Sci. Total Environ..

[32] Jia Z., Conrad R. (2009). Bacteria rather than Archaea dominate microbial ammonia oxidation in an agricultural soil. Environ. Microbiol..

[33] Cai Y., Zheng Y., Bodelier P.L.E., Conrad R., Jia Z. (2016). Conventional methanotrophs are responsible for atmospheric methane oxidation in paddy soils. Nat. Commun..

[34] Vestergaard G., Schulz S., Schöler A., Schloter M. (2017). Making big data smart—how to use metagenomics to understand soil quality. Biol. Fertil. Soils.

[35] Caporaso J.G., Kuczynski J., Stombaugh J., Bittinger K., Bushman F.D., Costello E.K., Fierer N., Pena A.G., Goodrich J.K., Gordon J.I. (2010). QIIME allows analysis of high-throughput community sequencing data. Nat. Methods.

[36] Lan Y., Wang Q., Cole J.R., Rosen G.L. (2012). Using the RDP Classifier to Predict Taxonomic Novelty and Reduce the Search Space for Finding Novel Organisms. PLoS ONE.

[37] Barberán A., Bates S.T., Casamayor E.O., Fierer N. (2012). Using network analysis to explore co-occurrence patterns in soil microbial communities. ISME J..

[38] Hall B.G. (2013). Building Phylogenetic Trees from Molecular Data with MEGA. Mol. Biol. Evol..

[39] Schöler A., Jacquiod S., Vestergaard G., Schulz S., Schloter M. (2017). Analysis of soil microbial communities based on amplicon sequencing of marker genes. Biol. Fertil. Soils.

[40] Douglas G.M., Beiko R.G., Langille M.G.I., Beiko R.G., Hsiao W., Parkinson J. (2018). Predicting the Functional Potential of the Microbiome from Marker Genes Using PICRUSt. Microbiome Analysis: Methods and Protocols.

[41] Shabbir S., Faheem M., Ali N., Kerr P.G., Wu Y. (2017). Periphyton biofilms: A novel and natural biological system for the effective removal of sulphonated azo dye methyl orange by synergistic mechanism. Chemosphere.

[42] Li X., Xie Q., Chen S., Xing M., Guan T., Wu D. (2019). Inactivation of phosphorus in the sediment of the Lake Taihu by lanthanum modified zeolite using laboratory studies. Environ. Pollut..

[43] Bere T., Chia M.A., Tundisi J.G. (2012). Effects of Cr III and Pb on the bioaccumulation and toxicity of Cd in tropical periphyton communities: Implications of pulsed metal exposures. Environ. Pollut..

[44] Sultana M.-Y., Akratos C.S., Pavlou S., Vayenas D.V. (2014). Chromium removal in constructed wetlands: A review. Int. Biodeterior. Biodegrad..

[45] Tiwari S., Patel A., Prasad S.M. (2018). Kinetin alleviates chromium toxicity on growth and PS II photochemistry in Nostoc muscorum by regulating antioxidant system. Ecotoxicol. Environ. Saf..

[46] Espinoza-Quiñones F., Silva E., Almeida Rizzutto M., Palácio S., Módenes A., Szymanski N., Martin N., Kroumov A. (2008). Chromium ions phytoaccumulation by three floating aquatic macrophytes from a nutrient medium. World J. Microbiol. Biotechnol..

[47] Maine M.A., Suñe N., Hadad H., Sánchez G., Bonetto C. (2009). Influence of vegetation on the removal of heavy metals and nutrients in a constructed wetland. J. Environ. Manag..

[48] Lu Y.-Z., Chen G.-J., Bai Y.-N., Fu L., Qin L.-P., Zeng R.J. (2018). Chromium isotope fractionation during Cr(VI) reduction in a methane-based hollow-fiber membrane biofilm reactor. Water Res..

[49] Bere T., Tundisi J.G. (2011). Toxicity and sorption kinetics of dissolved cadmium and chromium III on tropical freshwater phytoperiphyton in laboratory mesocosm experiments. Sci. Total Environ..

[50] Wnuk E., Walkiewicz A., Bieganowski A. (2017). Methane oxidation in lead-contaminated mineral soils under different moisture levels. Environ. Sci. Pollut. Res..

[51] Bowman J.P., Sly L.I., Hayward A.C. (1990). Patterns of tolerance to heavy metals among methane-utilizing bacteria. Lett. Appl. Microbiol..

[52] De Marco P., Pacheco C.C., Figueiredo A.R., Moradas-Ferreira P. (2004). Novel pollutant-resistant methylotrophic bacteria for use in bioremediation. FEMS Microbiol. Lett..

[53] Alam M.A., Wan C., Zhao X.-Q., Chen L.-J., Chang J.-S., Bai F.-W. (2015). Enhanced removal of Zn^2+^ or Cd^2+^ by the flocculating Chlorella vulgaris JSC-7. J. Hazard. Mater..

[54] Pandey V.C., Singh J.S., Singh D.P., Singh R.P. (2014). Methanotrophs: Promising bacteria for environmental remediation. Int. J. Environ. Sci. Technol..

[55] Choi D.W., Do Y.S., Zea C.J., McEllistrem M.T., Lee S.-W., Semrau J.D., Pohl N.L., Kisting C.J., Scardino L.L., Hartsel S.C. (2006). Spectral and thermodynamic properties of Ag (I), Au (III), Cd (II), Co (II), Fe (III), Hg (II), Mn (II), Ni (II), Pb (II), U (IV), and Zn (II) binding by methanobactin from Methylosinus trichosporium OB3b. J. Inorg. Biochem..

[56] Tsutsumi M., Kojima H., Fukui M. (2012). Vertical profiles of abundance and potential activity of methane-oxidizing bacteria in sediment of Lake Biwa, Japan. Microbes Environ..

[57] Mayr M.J., Zimmermann M., Guggenheim C., Brand A., Bürgmann H. (2020). Niche partitioning of methane-oxidizing bacteria along the oxygen–methane counter gradient of stratified lakes. ISME J..

[58] Van Grinsven S., Sinninghe Damsté J.S., Abdala Asbun A., Engelmann J.C., Harrison J., Villanueva L. (2019). Methane oxidation in anoxic lake water stimulated by nitrate and sulfate addition. Environ. Microbiol..

[59] Xavier J.C., Costa P.E.S., Hissa D.C., Melo V.M.M., Falcão R.M., Balbino V.Q., Mendonça L.A.R., Lima M.G.S., Coutinho H.D.M., Verde L.C.L. (2019). Evaluation of the microbial diversity and heavy metal resistance genes of a microbial community on contaminated environment. Appl. Geochem..

[60] Su J.F., Zhang H., Huang T.L., Wei L., Li M., Wang Z. (2019). A new process for simultaneous nitrogen and cadmium(Cd(II)) removal using iron-reducing bacterial immobilization system. Chem. Eng. Process.—Process Intensif..

[61] Zhang B., Zhang J., Liu Y., Shi P., Wei G. (2018). Co-occurrence patterns of soybean rhizosphere microbiome at a continental scale. Soil Biol. Biochem..

[62] Wu H., Li Y., Zhang W., Wang C., Wang P., Niu L., Du J., Gao Y. (2019). Bacterial community composition and function shift with the aggravation of water quality in a heavily polluted river. J. Environ. Manag..

[63] Radková A.B. (2019). The role of secondary oxides in potentially toxic elements migration. Int. Multidiscip. Sci. GeoConf. SGEM.

[64] Zolgharnein H., Karami K., Assadi M.M., Sohrab A.D. (2010). Molecular characterization and phylogenetic analyses of heavy metal removal bacteria from the Persian Gulf. Biotechnology.

[65] Chen L.-X., Li J.-T., Chen Y.-T., Huang L.-N., Hua Z.-S., Hu M., Shu W.-S. (2013). Shifts in microbial community composition and function in the acidification of a lead/zinc mine tailings. Environ. Microbiol..

[66] Vorobev A., Jagadevan S., Baral B.S., DiSpirito A.A., Freemeier B.C., Bergman B.H., Bandow N.L., Semrau J.D. (2013). Detoxification of mercury by methanobactin from Methylosinus trichosporium OB3b. Appl. Environ. Microbiol..

