# Supplementary material for: Enhanced Adsorptive Bioremediation of Heavy Metals (Cd2+, Cr6+, Pb2+) by Methane-Oxidizing Epipelon
Source: Microorganisms. 2020 Apr 1;8(4):505. doi: 10.3390/microorganisms8040505 (PMC7232255; doi:10.3390/microorganisms8040505)
Supplement: Supplementary file 1 [file microorganisms-08-00505-s001.pdf]

Enhanced Adsorptive Bioremediation of Heavy Metals (Cd^{2+}, Cr^{6+}, Pb^{2+}) by Methane-Oxidizing Epipelon

Muhammad Faheem[1,2], Sadaf Faheem[3], Jun Zhao[1], Philip G Kerr[4], Nasrin Sultana [1,2], and Zhongjun Jia[1*]

[1]State Key Laboratory of Soil and Sustainable Agriculture, Institute of Soil Science, Chinese Academy of Sciences, Nanjing, 210008, Jiangsu Province, P.R. China

[2]University of Chinese Academy of Sciences, Beijing, 100049, China

[3] College of Environment, Hohai University, 1 Xikang Road, Nanjing, China

[4]School of biomedical Science, Charles Sturt University, Wagga Wagga, NSW 2678, Australia.

*Corresponding author: Dr Zhongjun Jia

Email: jia@issas.ac.cn

This SOM file contains:

Supplementary Table S1 to S4

Supplementary Figure S1 to S5

Supplementary References

Table S1. Primers and PCR conditions used in this study

Primer name	Primer sequence (5'-3')	Targeted gene	Thermal condition	Molecular analysis	Reference
**515F**	GTGCCAGCMGCCGCGG	universal 16S rRNA gene	95 °C, 5 min; 33× (95 °C, 30 s; 54 °C, 30 s; 72 °C, 30 s); 72 °C, 8 min	MiSeq sequencing	[1]
**907R**	CCGTCAATTCMTTTRAGTTT				
**A189F**	GGNGACTGGGACTTCTGG	*pmoA* gene	95 °C, 3 min; 35× (95 °C,10 s; 54 °C, 30 s; 72 °C, 30 s; 80 °C, 5 s; plate read); melt curve 65 °C to 95 °C, incremental 0.5 °C, 0:05+plate read	Real-time PCR	[2,3]
**mb661r**	CCGGMGCAACGTCYTTACC		95 °C,5 min; 35 95 °C,30s; 54 °C, 30 s; 72 °C, 45 s); 72 °C, 10 min	MiSeq sequencing	

Table S2. Summary of MiSeq amplicon sequencing of 16S rRNA genes from total DNA sample

**Treatment**	**High Quality Read Number**		
	**Replicate-1**	**Replicate-2**	**Replicate-3**
**6 ppm $^{13}CH_4$**	46980	48980	51980
**120000 ppm $^{13}CH_4$**	54713	571157	55654
**Cr+120000 ppm $^{13}CH_4$**	23343	27124	24463
**Pb+120000 ppm $^{13}CH_4$**	55454	66328	56110
**Cd+120000 ppm $^{13}CH_4$**	43119	45264	40069

Table S3. Summary of Illumina MiSeq amplicon sequencing of *pmoA* genes from total DNA

Treatment	High Quality Read Number	
	**16S rRNA gene**	***pmoA* gene**
120000 ppm $^{13}CH_4$	59575	17145
Cr+120000 ppm $^{13}CH_4$	21432	5600
Pb+120000 ppm $^{13}CH_4$	58616	14521
Cd+120000 ppm $^{13}CH_4$	55432	12518

Table S4 Topological properties of the co-occurrence networks of lake microbial communities

Network parameter	Cr+120000 ppm $^{13}CH_4$	Pb+120000 ppm $^{13}CH_4$	Cd+120000 ppm $^{13}CH_4$	120000 ppm $^{13}CH_4$	6 ppm $^{13}CH_4$
**Nodes**	156	256	269	287	178
**Edges**	2128	4228	4526	5128	2728
**Average Degree**	1.11	1.65	1.71	1.87	1.23
**Network diameter**	10	10	10	10	10
**Graph density**	0.0952	0.166	0.173	0.182	0.097
**Modularity**	0.17	0.18	0.19	0.193	0.181
**Network distance**	1.65	2.23	2.34	2.54	1.88

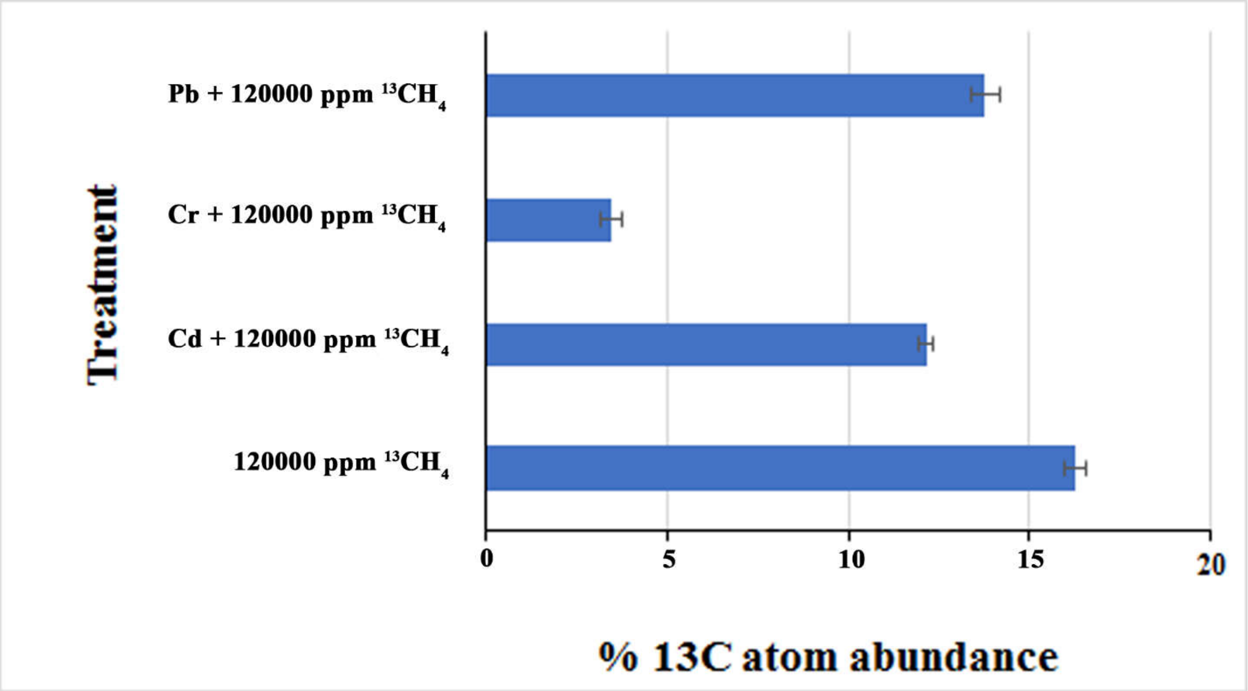

Fig S1. Percentage of ^{13}C atoms abundance accumulated by methanotrophs in Epipelon

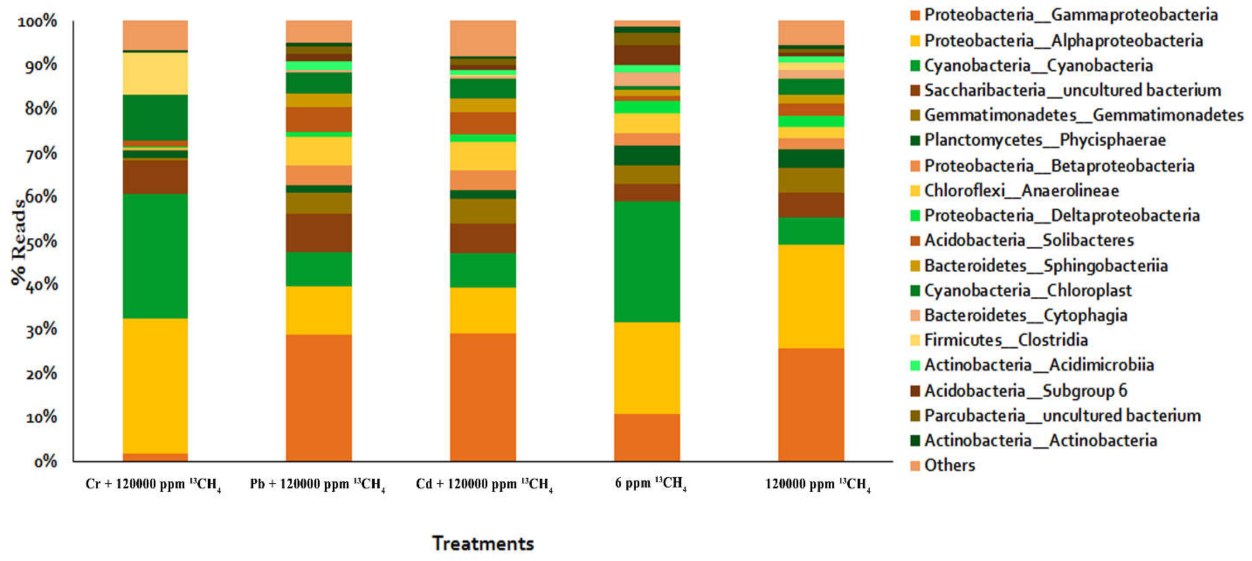

Figure S2. The changes in community composition of microbes following microcosm incubation with respective methane doses based on high throughput sequencing of 16S rRNA genes at class level.

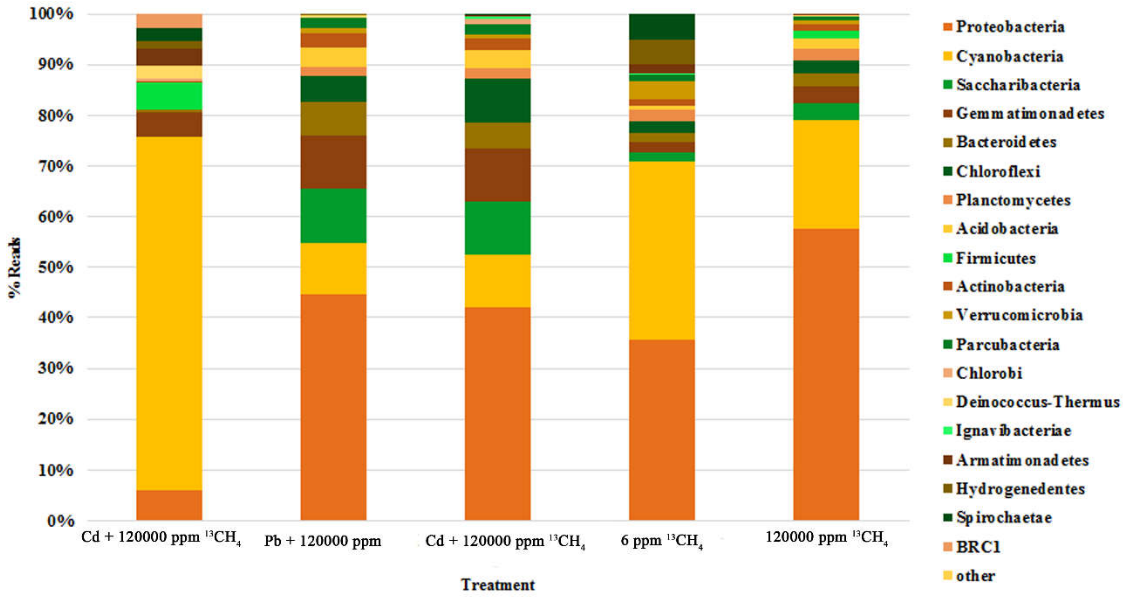

Figure S3. The changes in community composition of microbes following microcosm incubation with respective methane doses based on high throughput sequencing of 16S rRNA genes at phylum level.

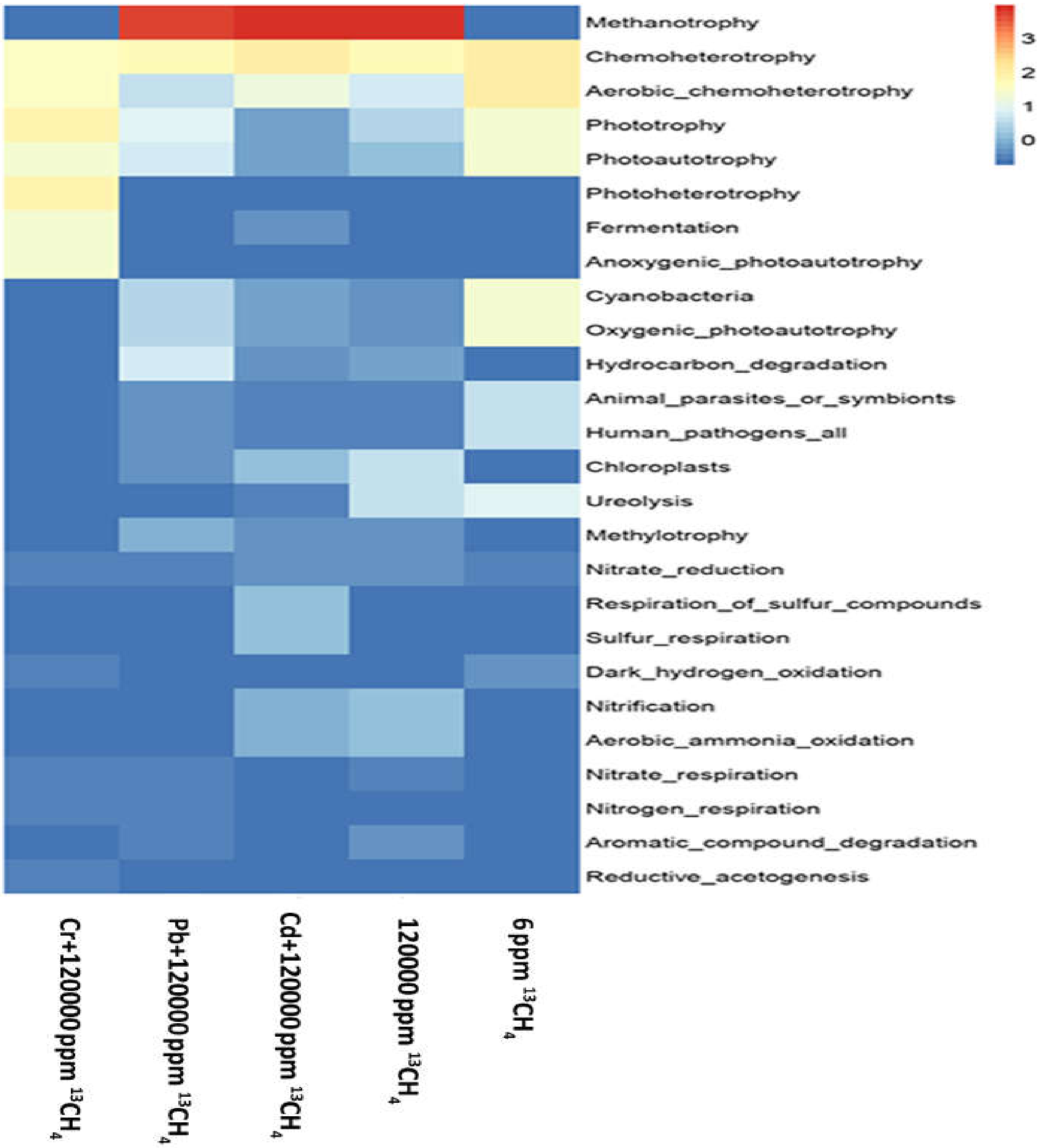

Figure S4. Functional pheat map of Epipelon under different methane and metals doses

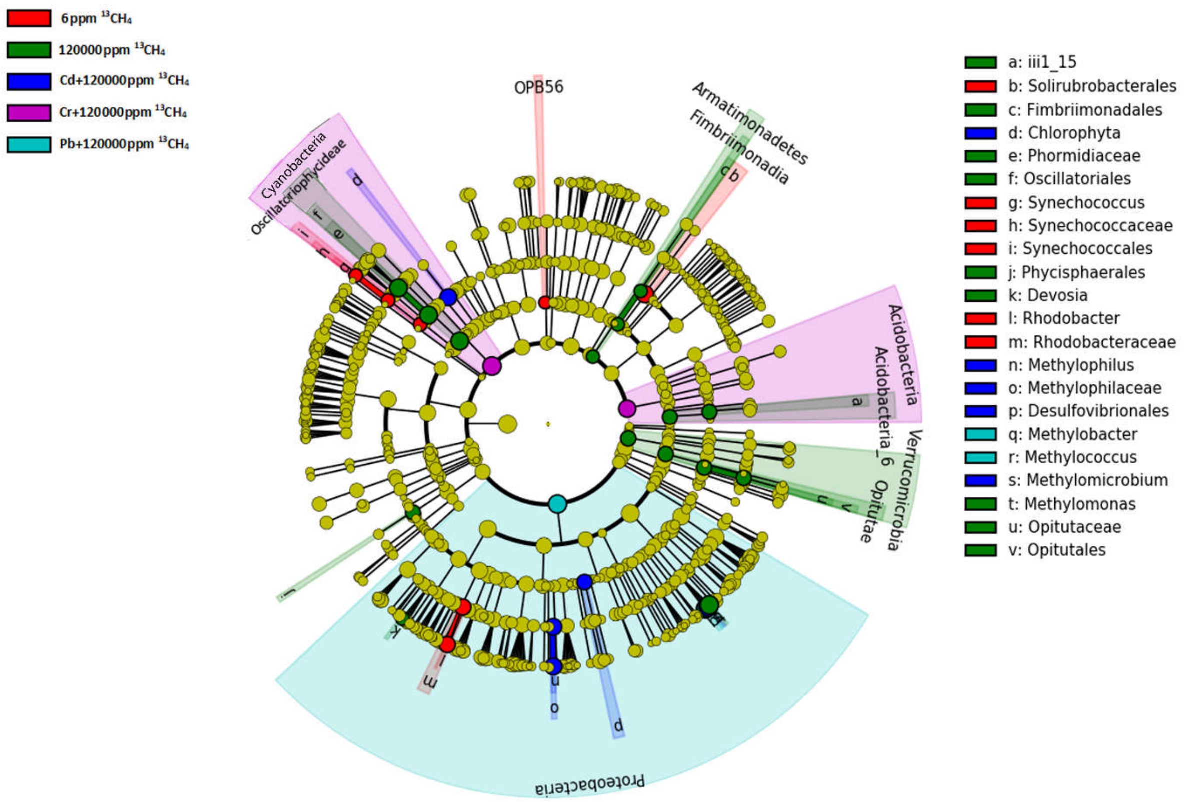

Figure S5. linear discriminant analysis effect size pipeline (LEfSe) analysis for dominant phyla, class and species.

References

1. Stubner, S. Enumeration of 16S rDNA of Desulfotomaculum lineage 1 in rice field soil by real-time PCR with SybrGreen™ detection. *Journal of Microbiological Methods* **2002**, *50*, 155-164, doi:https://doi.org/10.1016/S0167-7012(02)00024-6.
2. Costello, A.M.; Lidstrom, M.E. Molecular characterization of functional and phylogenetic genes from natural populations of methanotrophs in lake sediments. *Applied and environmental microbiology* **1999**, *65*, 5066-5074.
3. Holmes, A.J.; Costello, A.; Lidstrom, M.E.; Murrell, J.C. Evidence that particulate methane monooxygenase and ammonia monooxygenase may be evolutionarily related. *FEMS Microbiol Lett* **1995**, *132*, 203-208, doi:10.1016/0378-1097(95)00311-r.
